# Essay on Biomembrane Structure

**DOI:** 10.1007/s00232-019-00061-w

**Published:** 2019-03-15

**Authors:** Christoph Gerle

**Affiliations:** 1 Institute for Protein Research, Osaka University, Suita, Japan; 2 Core Research for Evolutional Science and Technology, Japan Science and Technology Agency, Kawaguchi, Japan

**Keywords:** Membrane structure, Membrane protein, Lipid bilayer, Singer–Nicolson, History of science, Structural biology

## Abstract

Of all the macromolecular assemblies of life, the least understood is the biomembrane. This is especially true in regard to its atomic structure. Ideas on biomembranes, developed in the last 200 years, culminated in the fluid mosaic model of the membrane. In this essay, I provide a historical outline of how we arrived at our current understanding of biomembranes and the models we use to describe them. A selection of direct experimental findings on the nano-scale structure of biomembranes is taken up to discuss their physical nature, and special emphasis is put on the surprising insights that arise from atomic scale descriptions.

## Introduction

The three key macromolecules of life are the oligomers of nucleic acids, that is DNA and RNA, oligomers of amino acids, that is proteins, and a multitude of lipids in the aggregate form of cell membranes. DNA and proteins are not only linked via the mRNA but also by the fact that both are linear polymers that assemble into 3D structures which consist of repeating units of only four kinds of nucleic acids or only 20 different kinds of amino acids. Biomembranes are set apart. These are no linear polymers; rather, they are composed of a wide diversity of many single amphiphilic lipid molecules forming a volume enclosing 3D structure whose architecture is not encoded in genetic information. Owing to this, the fact that biomembranes are as fundamental to life as DNA/RNA and proteins might be overlooked. And for a long time, it was doubted whether general statements describing common properties of the biomembrane could be made as such. Despite their very different structures and compositions, the gross structure of all three macromolecules of life is determined by their amphiphilic nature and their chemical environment of liquid water. Together these minimize the decrease in entropy that is caused by the ordering of water through the exposure of their hydrophobic groups to bulk water. In other words, the presence of liquid bulk water surrounding the macromolecules of life gives the entropic term the decisive weight in the energetics that govern their overall structure—an important link for understanding them.

For understanding the properties of the macromolecules of life, atomic scale structures are key. Indeed, before the arrival of experimentally determined structures of DNA and proteins, ideas on how they might appear at the atomic scale were strongly influenced by X-ray structures of inorganic molecules such as crystalline salt [1]. Since the first experimentally derived atomic scale models of DNA and protein were reported 60 years ago [2–5], many thousands of atomic coordinates have been deposited in the protein data bank archives [6] (wwPDB; http://www.pdb.org), which currently contain more than 149,000 entries. However, in stark contrast to this impressive body of knowledge, relatively little is known about the structure of biomembranes: not only are there only several hundred known unique membrane protein structures (Membrane Proteins of Known 3D Structure; http://blanco.biomol.uci.edu/mpstruc/) [7], but also is the rate of their discovery much slower than anticipated (see growth curve in [7]). Importantly, very few of the deposited structures include a full embedding lipid bilayer and thus far not a single membrane protein structure has been determined under physiological membrane potential. As an example of how difficult it is to know and understand even a seemingly simple geometric property of biomembranes, the question of how thick they are will act as a leitmotif for this essay.

## Historical Background

Not long after Matthias Schleiden and Theodor Schwann had proposed in 1838 that the basic unit of all life on our planet is the cell [8, 9], Moritz Traube put forward the idea that cells must be surrounded by a semi-permeable barrier. Then, at the turn of the last century, studies on the effectiveness of anesthetics by Meyer and Overton suggested that this barrier might be formed by lipidic substances such as lecithin and cholesterol [10, 11]. Work on the structurally simple red blood cells which lack internal membranes proved to be productive, and in 1925 surface area measurements of extracted phospholipids spread on a Langmuir trough gave the correct indication that the cell membrane is formed by a bilayer of lipids [12]. In the same year, Hugo Fricke measured the electric capacitance of red blood cells for estimating the thickness of their cell membrane [13]. Assuming a low dielectric constant *ε* for lipids of ~ 3, his calculations gave a value of 3.3 × 10^−7^ cm, i.e. 33 Å, for the thickness of the cell membrane—a value much thinner than anybody expected. Hugo Fricke did not take into account the fact that lipids have a hydrophilic head group of considerable size, and therefore, his experimental set-up did not measure the total thickness of the red blood cell membrane, but only that of its hydrophobic core. As a consequence, he compared the value of 33 Å with the length of lecithin measured in Langmuir trough monolayers, where the lipids take a non-physiological stretched conformation and concluded that red blood cell membranes consist only of a monolayer of lipids. Perhaps, it was this interpretational blunder that put Hugo Fricke’s experimental value of 33 Å on the sideline for many decades to come.

**Fig. 1:**
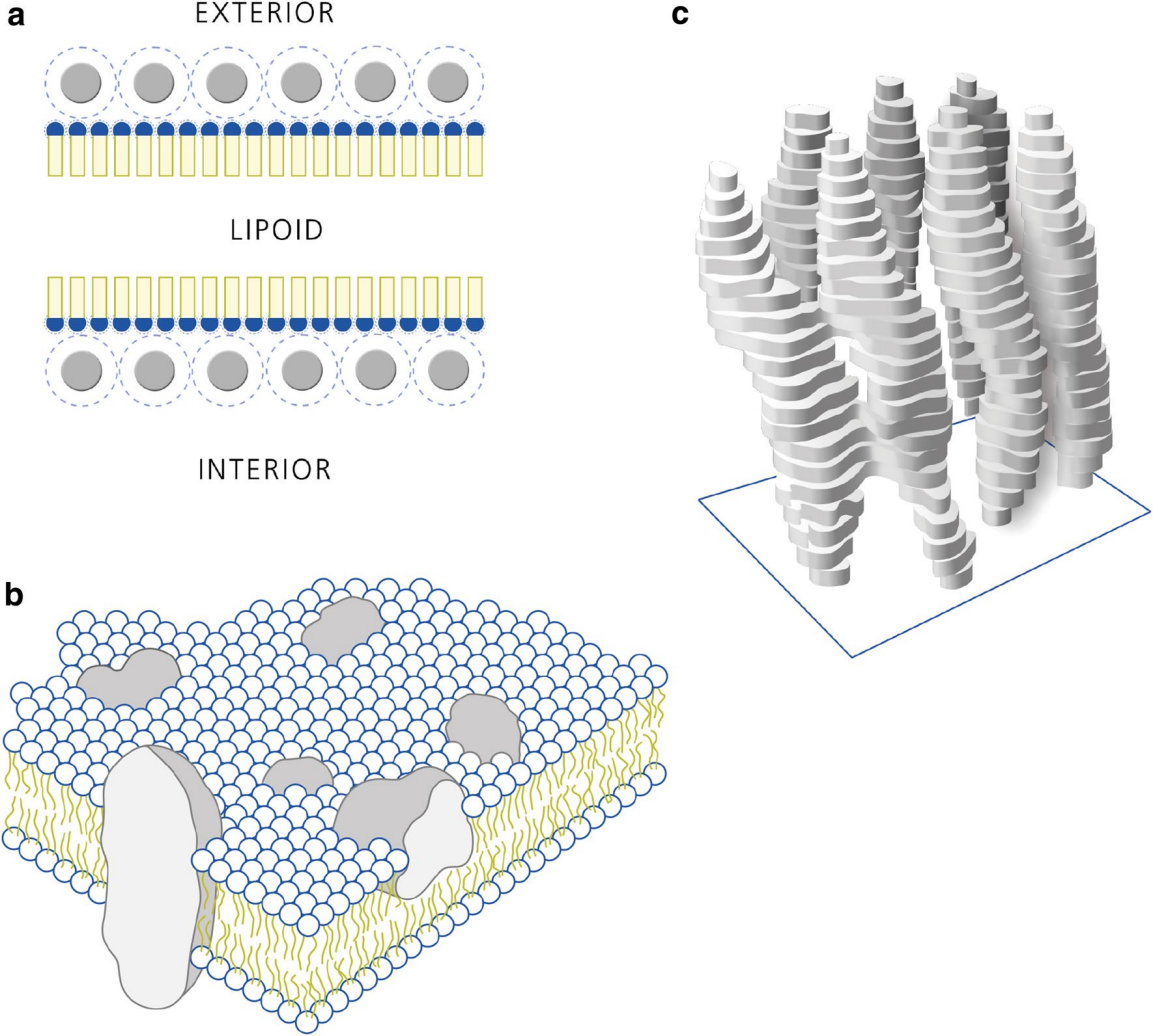
Models of the biomembrane. **a** The trilamellar membrane model by Davson and Danielli from 1935: a fluid core of lipidic substances is sandwiched by a bilayer of phospholipids whose hydrophilic headgroups (blue) are in electrostatic contact with a cask of water soluble globular proteins; (the membrane proteins) (redrawn after [14]). **b** Singer and Nicolson’s fluid mosaic model of the membrane from 1972 with a self-organized lipid bilayer acting as a passive matrix for transmembrane proteins freely floating in two dimensions (redrawn from [26]). See Table 1 for a list of model properties. **c** The first direct structural insight into membrane protein structure obtained by electron crystallography of the archaebacterial purple membrane in 1974 by Unwin and Henderson. Seven transmembrane alpha-helices demonstrate the concept of transmembrane proteins to be correct (redrawn from [31]). (Color figure online)

Davson and Danielli, who were the proponents of the very influential—albeit wrong in many aspects, as would eventually be shown—trilamellar membrane model, were aware of Hugo Fricke’s precise value of 33 Å [14], which clearly did not match their own estimation of around 120 Å for the thickness of biomembranes. Davson and Danielli’s trilamellar model would dominate textbooks for the next 40 years and thus influence basic thinking of life scientists around the world: according to this, the membrane consists of a bilayer of phospholipids sandwiching a disordered central layer of oily molecules such as cholesterol or fatty acids (Fig. 1a) [14]. Importantly, this lipid/oil sandwich was conceived to be unstable in itself and thus mainly held together by the strong, localized electrostatic interaction of a layer of water-soluble proteins on both sides. Thinking on biomolecules at the time of Davson and Danielli was profoundly influenced by the success of the Braggs in using X-ray diffraction to build models at the atomic scale for inorganic matter such as crystals of table salt [1]. These atomistic insights, together with the precise description of (biologically important) water–salt solutions by Debey-Hückel [15], gave the impression that local, directed strong electrostatic interactions between opposite charges such as salt-bridges, should govern the gross structure of the macromolecules of life. The trilamellar model was further endorsed by early electron micrographs of cell membranes obtained in the 1950s by the Romanian cell biologist Palade and the Danish biologist Sjörstrand [16, 17]; their micrographs of OsO_4_ fixated and negatively stained cells visualized features matching the predicted trilamellar structure. Importantly, the trilamellar structures found in the images also matched the expected thickness of ~ 120 Å for the cell membrane.

**Table 1:** A list of properties of the fluid mosaic model of the structure of biomembranes as described in Singer and Nicolson’s seminal 1972 paper [26]

Properties of the Singer–Nicolson membrane model	Finding, modification or extension
Gross structure is unitary	Confirmed
Gross structure is amphiphilic	Confirmed
Hydrophobic effect is major organizing force	Confirmed
Lipids take bilayer form	Confirmed
Lipids are fluid	Confirmed
Transmembrane proteins are common	Confirmed
Diffusion as main source of mobility in the membrane	Confirmed
Asymmetric protein insertion	Confirmed
Asymmetric lipid distribution	Confirmed
Membrane proteins are mostly globular	Confirmed
Membrane proteins are mostly alpha helical	Confirmed
Membrane thickness is ~ 70–90 Å	At ~ 45 Å thinner and a more narrow range of thickness
Mosaic distribution of membrane proteins	Super-assemblies and super-complexes appear to be common
Weak interactions between lipids and membrane proteins	Strong interactions can be found
No long range interaction (> 1/10 µm)	Long range re-modelling via super-complexes
Lipids exist only external of membrane proteins	Multisubunit membrane proteins often have high lipid content
2D lipid matrix is flat at nano-scale	Membrane bending and z-axis displacement at nano-scale
Random lateral distribution of lipids	Lipid rafts
Population of membrane proteins is mostly heterogeneous	Areas of homogenous protein populations are not rare

Properties are divided into ones that were firmly confirmed in the last decades and ones that experienced modification or extension

A further development of the dominant Davson and Danielli trilamellar membrane model was the concept, put forward by Robertson [18], that all biomembranes should have the same basic architecture. Considering the wide variety of lipids found in the many types of cells harbouring a plethora of different architectures of cell membranes and cell organelle membranes, this concept of the unit membrane, as it was called, is neither self-evident nor was it widely accepted [19]. Robertson also modified the water-soluble proteins that were thought to enforce the stability of the membrane from globular shape to a sheath of β-sheets forming a stabilizing cask that matched the smooth appearance of the membrane observed in the micrographs of Sjörstrand and Palade. Meanwhile in the quest for the structure of DNA, the trilamellar membrane model found its counterpart in Linus Pauling’s triple helix model of DNA structure. The triple helix model had its hydrophobic purines and pyrimidines pointing out into bulk solution and charged phosphate-sugars building the triple helix’ backbone were held together by the electrostatic interaction afforded through intercalating positive Mg^2+^ ions [20]—a model clearly influenced by ideas of strong and directed molecular interaction. Linus Pauling correctly predicted the structure of α-helices and β-sheets as important architectural elements of protein structure governed by the geometrically precise interactions of individual groups of atoms via hydrogen bonds [21, 22]. However, although local and directed chemical bonds determine the structure of organic molecules, non-directional entropic phenomena govern the gross structure of the macromolecules of life. Shortly after Linus Pauling’s proposal, Watson and Crick, who knew of the triple helix model and the X-ray diffraction patterns taken by Rosalind Franklin and Raymond Gosling from calf thymus DNA [5], built the double helix model of DNA structure that would stand the test of time: hydrophobic bases facing inward on each other and the phosphate-sugar backbone facing bulk water [4]. A few years later, the first experimentally determined atomic scale 3D model of a macromolecule of life, the whale protein myoglobin at atomic resolution, was presented by the Kendrew/Perutz team in Cambridge [2]. Though seemingly chaotic at first glance, the α-helical substructure proposed by Pauling had been found. But perhaps even more consequential was the observation that “[...] non-polar residues make up the bulk of the interior of the molecule” and that polar residues dominate the surface [23]. The following years of pioneering work on the structure of DNA and water soluble proteins laid the groundwork for thermodynamic considerations of what governs their overall structure. This culminated in the general concept of the hydrophobic effect that stabilizes the gross structure of DNA and proteins in liquid water [24, 25].

### The Fluid Mosaic Model of the Biomembrane

It was Singer and Nicolson’s insight that the same is true for the third class of amphiphilic macromolecules of life. An insight, which freed the “classic” trilamellar model from its stabilizing sheath of water-soluble proteins and enabled the fluid mosaic model (Fig. 1b) [26]. In this radical new model of biomembrane structure, it is the entropic punishment of exposing hydrophobic lipid acyl chains to bulk water that ensures the stability of the passive 2D lipid bilayer matrix while allowing mobility of its active ingredient, the transmembrane or lateral bound membrane proteins. Singer and Nicholson posited their new unit membrane to have an expected thickness of ~ 70 to 90 Å; considerably thinner than the ~ 120 Å of the trilamellar membrane. The great conceptual advance and the lucid writing of Singer and Nicolson’s 1972 article made their beautifully drawn cartoon of the new membrane model one of the most influential science illustrations of the twentieth century. The drawing still appears virtually unaltered in all standard biochemistry textbooks and is a powerful meme [27] within the life science community. In the fluid mosaic model of the membrane, amphiphilic membrane proteins float randomly in the passive matrix of a fluid bilayer of amphiphilic lipids. These, by virtue of the self-organization induced by the hydrophobic effect, have their hydrophobic acyl chain facing inward and their hydrophilic headgroups facing bulk water (see Table 1 for a list of properties of the Fluid Mosaic Membrane Model from 1972). Though it is organized as a 2D matrix, Singer and Nicolson stressed the 3D nature of the biomembrane: both matrix lipids and membrane proteins have direction and an asymmetric distribution. Thus, in an important shift, Singer and Nicolson’s model broke with the symmetric depiction of all previous biomembrane models. This new image of the asymmetric biomembrane fitted well into novel concepts of the biomembrane acting as a permissible capacitor for electric signal transmission in electrophysiology [28], as well as the paradigm shift announced by Peter Mitchell’s formulation of bioenergetics based on proton gradients across asymmetric biomembranes as the pivotal energy intermediate in oxidative phosphorylation (OXPHOS) [29]. Since its publication the fluid mosaic model has been modified or extended in numerous publications that deal with one or several aspects where the model’s assumptions collide with experimental evidence (see Table 1 for an incomplete list). For instance, a review by Engelman from 2005 stressed on the patchiness and crowdedness of most cellular membranes which is in clear conflict to the image of free floating membrane proteins depicted in the cartoon of Fig. 1b [30]. Notwithstanding, most discussions on the subject, including this essay, still take place in the context established by Singer–Nicolson.

## Direct Experimental Insights into the Structure of the Biomembrane

### The Purple Membrane

**Fig. 2:**
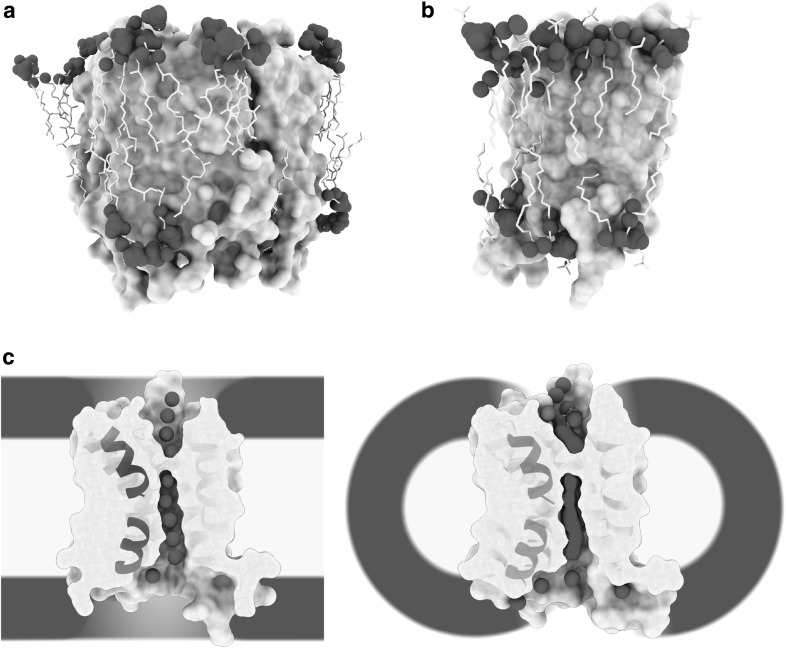
Atomic structures of membrane proteins in the biomembrane. **a** High-resolution analysis of the purple membrane by electron crystallography enabled the first view of a natural membrane at the atomic scale by direct structural methods [34, 35]. Protein in grey, lipid headgroup heteroatoms (phosphor, oxygen and nitrogen) as blue spheres and hydrophobic archaebacterial specific branched phytanoyl acyl chains in yellow. A non-annular lipid can be seen at the upper left edge (PDB: 1AT9). All lipids shown are of the natural membrane modelled according to the crystallographic density map. **b** Structure of the mammalian water channel aquaporin-0 in a bilayer of the synthetic phospholipid DMPC [36]. Note that membrane dimensions are very similar to the evolutionary distant purple membrane. Colour coding as in **a**; (PDB: 2B6O). All lipids shown are of the reconstituted membrane modelled according to the crystallographic density map. **c** Cut-through view of the mammalian brain water channel aquaporin-4 determined in the context of a full lipid bilayer by electron crystallography (left panel) [37] and in the context of detergent micelles by X-ray crystallography (right panel) [41]. Positions of water molecules are sharply defined (left panel) or smeared out (right side), possibly as a consequence of a dielectric constant-dependent change in the strength of the dipole moment of two short alpha-helices (depicted as ribbon diagrams). Drawing based on [42]. Colour coding as in **a** (PDB: 3IYZ and 3GD8). Positioning of the cartoon membrane or detergent micelle relative to the membrane protein is based on the crystallographically determined position of the non-natural reconstituted membrane. (Color figure online)

Finally, in 1975, the first direct experimental visualization of a membrane protein in the context of its physiological membrane was achieved: bacteriorhodopsin of the natural highly ordered 2D crystal of the purple membrane of the archaebacteria *Halobacterium halobium* (Fig. 1c) [31]. Though limited to about 7 Å resolution, the 3D reconstruction obtained by electron crystallography clearly showed bacteriorhodopsin to span the total of the membrane and thus beautifully demonstrated Singer and Nicolson’s concept of transmembrane, amphiphilic membrane proteins to be correct. At the same time, the purple membrane’s highly ordered, tight arrangement of bacteriorhodopsin membrane proteins—the very reason it was possible to use electron crystallography to analyse its structure—made the first experimental modification to the concept of free floating membrane proteins. In fact, it is only recently that the new technique of high speed AFM made it possible to recognize that bacteriorhodopsin is not only too tightly packed to float freely in the membrane, but also that light-induced movements of the bacteriorhodopsin trimer are possibly coordinated across the whole purple membrane via long range interactions [32]. In their seminal 1975 study of the purple membrane by electron crystallography, Henderson and Unwin measured the purple membrane’s thickness to be ~ 45 Å, which is considerably thinner than the 70–90 Å that Singer and Nicolson had predicted. It took another 22 years and the development of a cryo-electron microscope equipped with a liquid helium-cooled specimen stage [33] to improve the 3D map derived from electron crystallography to a level sufficient to yield atomic coordinates for both protein and lipid constituents of the purple membrane (Fig. 2a) [34, 35]. This afforded the first precise measurement of the thickness of a physiological biomembrane: total thickness 42 Å and thickness of the hydrophobic core 30 Å. After a 70-year hiatus, Hugo Fricke’s value of 33 Å for the thickness of the red blood cell’s membrane suddenly seemed to be realistic. However, since the archaebacterial purple membrane from the extremophile *Halobacterium halobium* has a cell membrane very different from that of mammals, the universality of the findings was not clear. For example, it includes exotic components such as methyl group branched phytanoyl lipid chains (chain length C16), which is specific to archaebacteria, as a major component.

### Water Channels in the Membrane

Following these observations, in 2005, another set of atomic coordinates was reported for a membrane protein as flat as bacteriorhodopsin, together with its embedding membrane composed of the synthetic lipid DMPC (Fig. 2b) [36]. Again, this was achieved by electron crystallography making use of the same liquid helium-cooled cryo-electron microscope; but in this instance, the membrane protein was the mammalian eye lens protein aquaporin-0, a member of the water channel family extracted from the eye lens of New Zealand sheep. The lipid was a relatively short (chain length C14) synthetic phosphocholine, yet the dimensions of the membrane were astonishingly similar to those of the archaebacterial purple membrane with a total thickness of ~ 50 Å and a hydrophobic core of ~ 28 Å. A similar membrane thickness was indicated by the visualization of synthetic lipids from both leaflets in direct contact with the membrane protein in 2D crystals of rat aquaporin-4, a water channel found in ganglia cells of the brain [37]. Perhaps these findings are an indication of an evolutionary pressure to conserve a membrane thickness of ~ 50 Å and a hydrophobic core of ~ 30 Å: a strong case for Robertson’s idea of a unit membrane with similar dimensions across all kingdoms of life.

The biological role of aquaporins is to facilitate the fast (~ 10^9^/s) transfer of water molecules across cell membranes while preventing protons from crossing the membrane. However, water has the ability to form proton wires via the Grotthus mechanism, which is biologically important for membrane proteins involved in proton pumping such as the mammalian cytochrome c oxidase, for example [38]. For this reason, the avoidance of proton transport in the presence of a chain of transmembrane water molecules is very surprising. A suggestion of how this proton exclusion might work while allowing very fast water transfer across the membrane was based on the first atomic model of a water channel, the water channel aquaporin-1 isolated from human red blood cells [39]. In this model, the dipole moment of two short alpha helices, tilted and fully immersed in the transmembrane region of the water channel, re-orients water molecules passing through the channel at its centre such that any potential proton wires are disrupted by breaking the continuous inter-water hydrogen bonding between the bulk water phases separated by the membrane. In this context, it is important to note that the strength of the effective alpha helical dipole moment depends on the dielectric constant *ε* of its chemical environment, with strength increasing for lower *ε* values (bulk water *ε* = ~ 80; lipidic core of a membrane *ε* = ~ 2). This results in a relatively strong dipole moment for short helices immersed in the hydrophobic core of the membrane [40]. The idea that an electric field is involved in breaking the proton wire was given a boost by high-resolution structures of aquaporin-4 determined in the membrane by electron crystallography [37] and in detergent micelles by X-ray crystallography (Fig. 2c) [41]. Despite the protein structures being almost identical, the water position for the structure solved in the low *ε* environment of a full lipid bilayer were sharply defined, whereas in the high *ε* environment of the detergent micelle they were smeared out [42]. Tilted, short alpha helices in the transmembrane region have also been reported for numerous ion channels where they appear to point at the channel traversing ions [43–45]. Interestingly, highly tilted transmembrane alpha-helices are also a hallmark of cation transporting rotary ATPases [46–51]. Electric fields are difficult to visualize at the nano-scale level of biological membranes; however, it seems that cells exploit the hydrophobic core of cell membranes to strengthen the electric dipole moment at the end of membrane immersed alpha helices and thus harness internal electric fields for functions such as coordinating water molecules or ions.

### Ion Channels in the Membrane

**Fig. 3:**
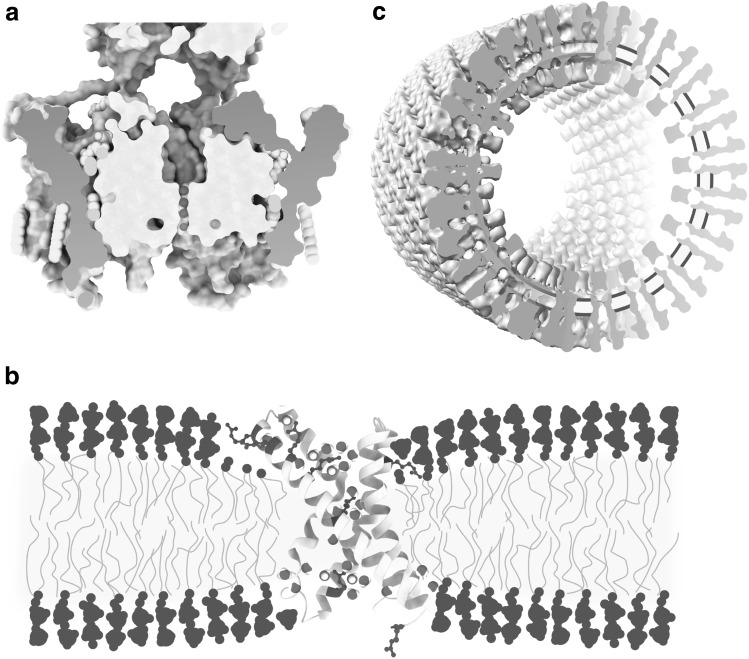
Ion channels in the biomembrane. **a** Cut-through view of a voltage sensing potassium channel [55]. Voltage sensor domains are physically separated from the ion channel domain by lipids of the embedding membrane and flexibly connected to the ion pore via extramembranous loops. Protein in grey with voltage sensor domain shaded in red, lipids in yellow, potassium ions in purple (PDB: 2R9R). Positioning of the reconstituted non-natural lipids is based on a crystallographic density map. **b** An isolated voltage sensor domain with wetted arginine residues and a locally deformed membrane. Protein in grey, ribbon diagram representation with arginines as blue ball and stick models, transmembrane water molecules in red/white. Drawing based on [54]. Positioning of the cartoon membrane relative to the voltage-sensor domain is based on results from neutron diffraction of reconstituted, non-natural membranes and molecular dynamic simulations. **c** Cut-through view of tubular crystals of the acetylcholine receptor from postsynaptic membranes of the electric organ from the Atlantic fish Torpedo marmorata. A rare close-up view of a natural cholesterol-rich membrane including large areas of non-annular lipids. For clarity, receptors in the right half of the cut-view are depicted schematically. The position of the membrane is indicated in yellow and blue. Drawing based on [59]. Positioning of the natural membrane relative to the ion channels is based on a density map obtained by cryo-electron microscopy. (Color figure online)

A well-known function of electric fields in biology is mediated by the voltage sensors attached to ion channels that sense changes in transmembrane potential for the gating of their respective ion channels, enabling the fast transmission of electric signals along the axons of neurons, for instance [52]. The strength of an electric field depends on the difference in electric potential and the combination of thickness and the value of the dielectric constant *ε* of the charge-separating insulator. At about 30 Å, the hydrophobic, insulating core of the cell membrane is already much thinner than was originally expected, enabling strong electric fields across the membrane at modest transmembrane potentials. Still, molecular dynamics simulation has suggested that in the close surroundings of voltage sensors (at the region where positively charged arginine residues enable the sensing of an electric field) the membrane is both wetted by the presence of water molecules and thinned beyond the usual ~ 30 Å (Fig. 3b) [54]. Given that water molecules confined to few molecule layers and restricted in their orientation have an exceptionally low dielectric constant of ~ 2 [53], the presence of water in the voltage sensor is unlikely to raise the local *ε* value. It has been proposed that the combination of wetting and membrane thinning results in the focusing of the electric field generated by the transmembrane potential onto the electric potential sensing charged arginine residues [54]. The inability to visualize voltage sensors in the context of a lipid bilayer experiencing a transmembrane potential, however, means that experimental verification of this proposal has not been possible yet. Still, in support of this proposal, visualization of individual lipid molecules sandwiched between ion pore and voltage sensor clearly showed that voltage sensors and ion pore do not form one tight protein entity in the membrane (Fig. 3a) [55]. Local manipulation of membrane thickness for focusing of the transmembrane electric field onto charged residues might not be limited to voltage sensors of ion conducting channels, but could include ion channel-independent, proton conducting voltage-sensing proteins [56].

The only case where an ion channel has been visualized at great detail in the same experimental data set as its physiological membrane is that of the helical tubes of the nicotinic acetyl-choline receptor which can be obtained from the postsynaptic membranes of the electric organ of the Atlantic fish *Torpedo* ray (Fig. 3c) [57]. The density map obtained by cryo-electron microscopy of the tubes, first by electron crystallography [58] and recently by real space averaging, allows the precise positioning of the natural lipid bilayer with its two hydrophilic headgroup regions and the hydrophobic core relative to the membrane spanning ion channel [59]. This provides a rare chance for direct measurement of the cell membrane dimension in the presence of a transmembrane protein which unlike bacteriorhodopsin or water channels is not flatly embedded in the membrane, but has large extramembranous hydrophilic domains immersed in bulk water on both sides of the membrane. Though not revealing atomic coordinates of individual lipids, the density map shows the bilayer to have a thickness of ~ 45 Å with a hydrophobic core of ~ 30 Å both at the site of contact with acetylcholine-receptors and the membrane region between them.

### Rotary ATPases Manipulate the Membrane

**Fig. 4:**
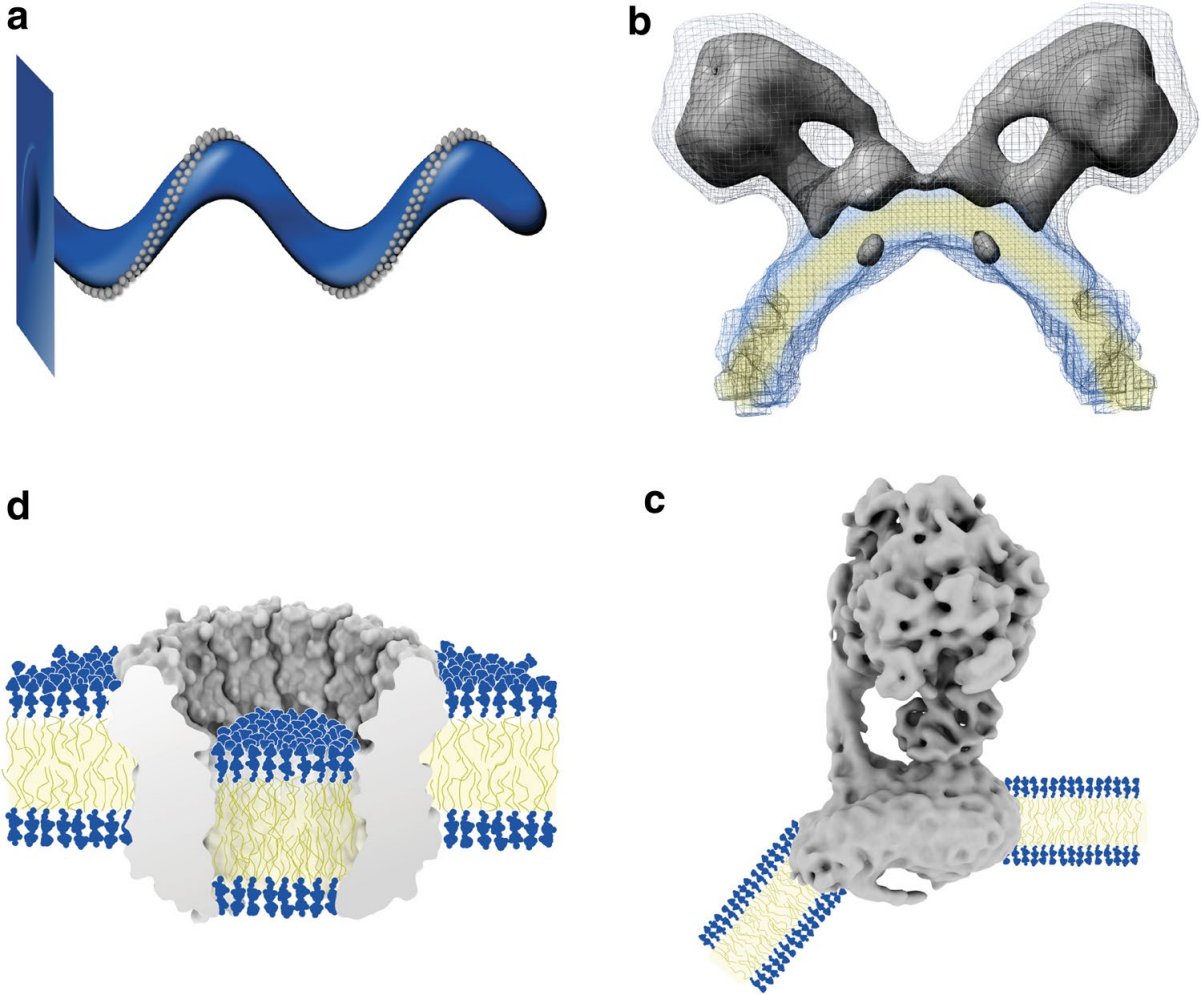
Rotary ATPases manipulate the biomembrane. **a** The first model proposing that rows of dimeric mitochondrial F-ATP synthases are shaping the architecture of the inner mitochondrial cristae membrane. Redrawn after [63]. The cartoon of a mitochondrial cristae was based on observations by freeze-fracture electron microscopy. **b** In situ electron tomographic analysis of the inner mitochondrial membrane from yeast showed that mitochondrial F-ATP synthase forms dimers at the high positive curvature edges of cristae [64] (EMDB: 2161). Position and shape of the indicated natural membrane in blue and yellow was obtained by direct structural analysis via subtomogram averaging of cryo-electron tomograms of the natural membrane. **c** Cryo-EM structures of the detergent solubilized and of the lipid bilayer reconstituted monomeric bovine F-ATP synthase demonstrated that the shape of the mammalian mitochondrial F-ATP synthase alone is sufficient to bend the membrane [66, 67] (EMDB: 3167). Position and shape of the cartoon membrane are based on cryo-electron tomograms of in vitro synthetic lipid reconstituted F-ATP synthase. **d** Cut-through view of the K-ring of the sodium pumping V-ATPase from *Enterococcus hirae* [60]. The luminal membrane is off-set relative to the embedding membrane by half a membrane thickness (PDB: 2BL2). Position and thickness of the luminal cartoon membrane are based on the crystallographically visualized natural luminal cardiolipin lipids, whereas the position of the surrounding cartoon membrane is based on the crystallographically visualized bound synthetic detergent molecules and bound sodium ions. (Color figure online)

A completely unexpected position for a natural lipid bilayer was found by X-ray crystallography of the sodium ion transporting K-ring of the bacterial V-ATPase from *Enterococcus hirae* (Fig. 4d) [60]. The bilayer visualized is not that of the membrane surrounding the K-ring, but the lipid bilayer occupying the lumen of this ring-shaped transmembrane protein. The inner diameter of the K-ring is wide enough to allow the presence of a number of cardiolipin lipids [61] large enough to form a full luminal lipid bilayer. Instead of being aligned to the height of the surrounding membrane, the luminal bilayer is shifted to the extracellular side by half the width of a hydrophobic membrane core, i.e. ~ 15 Å. This remarkable shift effectively leaves the lipid headgroups on the inside of the transmembrane ring at the height of the centre of the surrounding membrane, resulting in a thinning of the hydrophobic path between the ring lumen on the intracellular side and the extracellular region of the K-ring. Since V-ATPases, analogous to voltage sensors, are utilizing a transmembrane potential to fulfil their biological roles, shortening of the hydrophobic path between both sides of the membrane might have a role in stabilizing the assembly during rotary catalysis [62].

Though the K-ring structure shows that V-ATPases are capable of inducing a vertical shift of rotor ring luminal lipid bilayers in respect to their surrounding bilayer, as in all previous examples, V-ATPases are expected to reside in relatively flat areas of the membrane. In contrast, their molecular cousins in the family of rotary ATPases, the mitochondrial F-ATP synthases, have been shown to form long rows of dimers at the regions of high positive membrane curvature (Fig. 4a, b) [63, 64]. The ridges of the cristae of the inner mitochondrial membrane are the location where the transmembrane potential built up by the electron transport chain is used to generate ATP from ADP and P_i_ via rotary catalysis [65]. Oligomers of mitochondrial F-ATP synthase dimers are not just preferentially sequestered to regions of high positive curvature; rather, membrane bending is a property of the monomeric F-ATP synthase *per se*, as indicated by single particle cryo-EM of the detergent micelle solubilized bovine complex (Fig. 4c) [66] and conclusively demonstrated by cryo-electron tomography of 2D crystals of the monomeric bovine F-ATP synthase reconstituted into a synthetic lipid bilayer [67]. The clear tram-track features of the bilayer in the tomograms indicate that, while sharply bent by ~ 40°, bilayer thickness is preserved. A recent high-resolution cryo-EM structure of the transmembrane Fo domain of the mitochondrial F-ATP synthase from yeast suggests that the molecular mechanism by which the membrane is bent might be similar to that of BAR domain proteins [68]. BAR domain proteins, such as Endophilin A1, are capable to sense and induce positive membrane curvature. The molecular mechanism has been well studied, and it appears that it involves two independent effects: rigid electrostatic interaction with the headgroup region of the membrane, i.e. the kink of the BAR domain protein is forced upon the membrane, or by the insertion of amphiphilic alpha helices into the headgroup region of the membrane. In the latter mechanism displacing lipid headgroups induces the acyl chains of surrounding lipid molecules to enter the space below the inserted amphiphatic alpha helices and thus effectively reducing the local average lipid acyl chain volume in one leaflet of the membrane [69, 70]. The lack of an atomic model for the membrane bending subunit of the mitochondrial F-ATP synthase disallows deciding which type of mechanism is pre-dominant and highlights the need for membrane protein structures in the context of their embedding membrane. An unexplained interesting feature of cryo-EM studies by single particle reconstruction of the dimeric form from yeast mitochondria is the unassigned density at the monomer–monomer interface on the matrix side of the complex [71]. It is unclear whether this density stems from protein or lipid headgroups, or perhaps from detergent that was used to solubilize the membrane protein from its native membrane. In case this density stems from lipid headgroups of a lipid monolayer bridging opposing monomers of a dimer, it will be the first such case in membrane protein biology. And if true, this might be of consequence for the proposal that dimeric mitochondrial F-ATP synthases can form the mitochondrial permeability transition pore [72–74].

### The Active Membrane

**Fig. 5:**
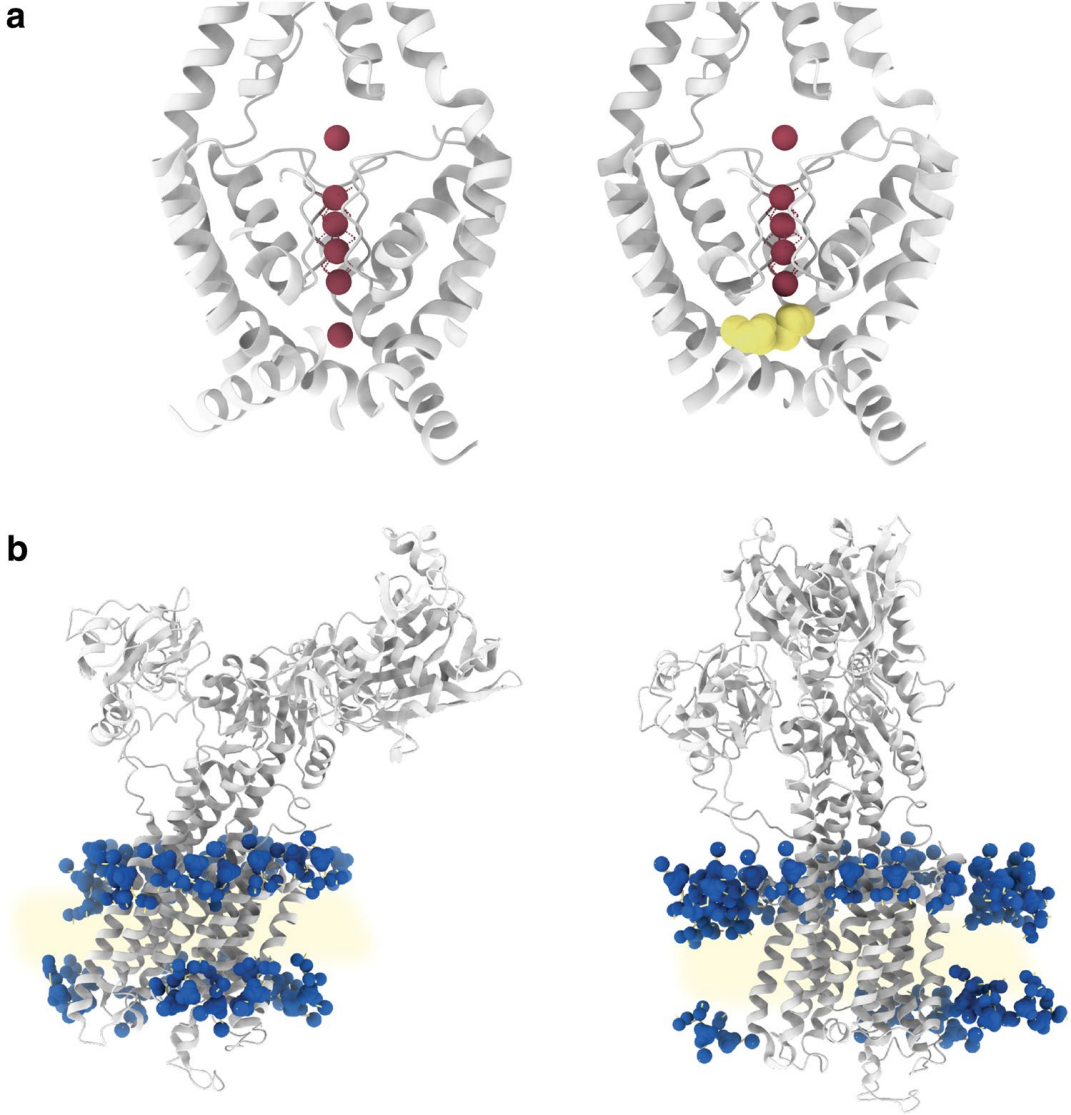
The active biomembrane. **a** X-ray crystal structures of the mechanical stress sensing human TRAAK potassium channel in open (left) and closed (right) conformation demonstrated gating of the ion pore to be achieved by a lipid acyl chain entering through a lateral crevice [75, 76]. Protein in grey, potassium ions in purple, and acyl chain in yellow (PDB: 4WFF and 4WFE). **b** Large-scale movements of the transmembrane domain of the mammalian SERCA calcium pump depicted in grey ribbons are accommodated through rocking motions in the membrane of relatively constant thickness [81] (PDB: 5XA7 and 5XA8). The position of lipid headgroup phosphor atoms of the reconstituted synthetic bilayer indicated by blue spheres was crystallographically determined. (Color figure online)

As described thus far, seen through the lens of high resolution structural studies the membrane takes a passive role. This is in line with the image of a matrix as proposed by Singer and Nicholson, which exerts its functional role chiefly through its composition, form and size. In contrast to this image of a mere matrix, structures of the TRAAK channel have shown that, while remaining chemically passive, the hydrophobic acyl chains of lipids can play a decisive active role in signal transduction (Fig. 5a) [75, 76]. The closing and opening of ion channels is mainly achieved through movement of the protein entity of the channel itself. This movement is a reaction to conformational changes induced by ligand binding, change in transmembrane voltage, or mechanical force exerted on it through the membrane or contacts with intracellular protein. For the TRAAK potassium ion channel, however, X-ray structures of its open and closed state visualized the entering of an individual acyl chain through lateral crevices in the transmembrane domain, blocking the ion pathway in the channel’s closed state. This is a surprising demonstration that lipids not integral to the channel itself can be the active agent in ion channel gating.

X-ray crystallography of 3D crystals of membrane proteins usually does not visualize the physiological environment of the lipid bilayer. This is due to the fact that most 3D crystals are grown from detergent-solubilized membrane proteins in the absence of a lipid bilayer. This is also true for type I 3D crystals consisting of stacked layers of membrane proteins in lipid bilayers, because the lipids present in type I 3D crystals are too disordered to be resolved by analysis of the X-ray diffraction pattern, which originates only from the ordered portion of the 3D crystal. Type I 3D crystals of the Ca^2+^ pump SERCA, a P-type ATPase, from the endoplasmic reticulum of rabbit muscle tissue have been used to solve high-resolution structures of many conformations of the enzyme’s pumping cycle [77]. The assumption that type I 3D crystals of stacked layers of SERCA do indeed contain a lipid bilayer had been confirmed by electron microscopy of thin 3D crystals [78]. Since SERCA’s Ca^2+^ pumping cycle involves large conformational changes in hydrophobic transmembrane alpha helices perpendicular to the assumed membrane plane, i.e. apparently into bulk water, it was expected that the local membrane thickness would adjust itself during the reaction cycle to accommodate the transmembrane movement [79, 80]. Recently, application of solvent contrast modulation X-ray crystallography allowed visualization of the lipids surrounding SERCA in the type I 3D crystal [81], and this approach was used to re-determine several structures of the pumping reaction cycle. By aligning the newly determined structures along the membrane plane, delineated by the phosphor atoms of the lipid headgroups, it became clear that it is not the membrane that adjusts its thickness to accommodate the large transmembrane movements. It is rather the SERCA Ca^2+^ pump that adjusts itself in the membrane of a relatively constant hydrophobic thickness of ~ 31–33 Å, by tilting as a whole and thus undergoing rocking motions during the pumping cycle (Fig. 5b). In this way, the exposure of hydrophobic alpha helices to bulk water during the pumping cycle is avoided. In other words, the membrane thickness dictates the membrane protein’s tilt angle in the membrane more than the membrane protein dictates the thickness of the membrane. Another surprising finding was the active role of lipids in the pumping cycle. Unlike the case of the TRAAK channel, this does not occur via acyl chains, but via interaction of lipid headgroups making electrostatic contacts with charged amino acid residues apparently aiding the movement of the reaction cycle. Thus far, most experimental approaches that visualize membrane proteins are unable to detect individual lipids. Therefore, it might turn out that lipids do play a much more active role than presently suspected.

## New Tools

New approaches such as solvent contrast X-ray crystallography as applied to the type I 3D crystals of the Ca^2+^ pump SERCA bring immensely valuable, novel insights into membrane biology; and their results are almost always surprising. Regrettably, though it has become more common with the maturation of the lipid cubic phase approach, growing large type I 3D crystals is far from trivial. Likewise, highly ordered 2D crystals, either naturally occurring like the purple membrane or grown in vitro like aquaporin-0, are very rare. Consequently, the statistics for atomic coordinates of membrane proteins with full lipid bilayers are very poor, with perhaps not more than 7 entries in the protein data bank versus a total number of more than 149,000; of those 7, none was obtained under physiological non-equilibrium conditions, that is, structure determination always occurred in the absence of a membrane potential. Clearly the age of structural membrane biology has hardly begun and new tools are necessary to allow an atomistic view on the biomembranes themselves. An exciting new approach towards structure determination of membrane proteins in their physiological environment of the membrane is realized by single particle cryo-EM of membrane proteins reconstituted into the lipid bilayer of nano-discs. This allows visualization of functionally important lipids together with the membrane protein [82]. Furthermore, strategies that combine liposomes with reconstituted membrane proteins and single particle cryo-EM might yield structures of integral membrane proteins under truly physiological conditions [83, 84]. However, reaching a resolution that is high enough to visualize lipids together with the membrane proteins will be challenging. Perhaps in the long run, cryo-electron tomography of planar lipid bilayers under non-equilibrium conditions will be the best option after the remaining technological challenges of data collection and image analysis of cryo-electron tomography have been solved. Likewise, for probing the thickness of natural biomembranes at the nano-scale in situ cryo-electron tomography is prone to be the best technique with interesting results already at hand [85, 86]. In high-resolution structures of integral membrane proteins determined by X-ray crystallography or cryo-EM, the correct assignment of lipids, even when tightly bound, is often error-prone due to the mobility of their headgroups. And less tightly bound lipids interacting with integral membrane proteins are usually lost during detergent mediated membrane protein purification. Thus, though desirable, most lipid–protein interaction is out of reach for direct structural methods. A feasible way out of this conundrum is the combination of molecular dynamic simulations (MD) with insights from X-ray crystallography. This approach is exemplified by the above-described results on SERCA or also by the first in molecular dynamic simulations predicted and then later by X-ray crystallography demonstrated interaction of PIP_2_ lipids with the Kir potassium ion channel [87, 88]. Very recently, a novel powerful approach in native mass spectrometry of membrane complexes allowed their direct ejection from native membranes [89]. This completely avoids the artefacts usually associated with detergent-based purification and thus allows to detect lipid species that are only weakly bound to integral membrane proteins. In combination with molecular dynamic simulations, this now opens up a completely new window on probing weak interactions of lipids with protein complexes in the native membrane as was demonstrated here on the interplay of the mitochondrial adenine nucleotide translocase (ANT-1) with fatty acids [89]. Finally, atomic scale insights into whole assemblies of membrane proteins in membranes of native composition will likely stay accessible only to in silico methods as shown recently for an ensemble of GPCRs in a membrane of physiological composition and asymmetry [90].

## Why ~ 30 Å?

A recurring theme in this essay is the thickness of the biomembrane, in particular of its hydrophobic core. It was shown that, against the expectations of the vast majority of biologists, the biomembrane is much thinner than was anticipated before the arrival of the first “hard structural data” in Henderson and Unwin’s seminal study on the purple membrane. As it turns out, Hugo Fricke’s measurement of 33 Å for the low *ε* portion of the red blood cell membrane was quite accurate. As noted before, with more than 40,000 lipid species, biomembranes are built up of an extremely diverse set of molecular compounds (http://www.lipidmaps.org/data/). Nevertheless, the examples of direct structural insights into biomembrane situated transmembrane proteins described in this essay suggest that the thickness of their hydrophobic core is astonishingly constant (see Table 2 for a list of thickness values reported in the literature discussed in this essay). And endowed with specific function when deviating from this standard thickness. Naturally, given that the examples discussed here are few and the lipids visualized mostly annular to the embedded membrane proteins, the generalization of the precise thickness of the hydrophobic core of the biomembrane has to be taken with caution. It should also be noted that, of the atomic structures discussed, only that of the lipids in the purple membrane are of a natural membrane. All other are that of reconstituted membranes of selected lipids. In addition, the usually high cholesterol content of plasma membranes is reflected only in the vesicular tubes grown from the natural postsynaptic membranes of the fish *Torpedo* ray. Interestingly, the tubes apparently contain cholesterol enriched regions in their outer membrane leaflet, possibly aiding acetylcholine receptors’ gating [59]. The likely important interaction of acetylcholine receptors and cholesterol in membranes of mixed lipid content was recently explored by coarse-grained MD detecting a clear de-mixing of lipid species and receptors [91]. Early X-ray and neutron diffraction studies on 40% cholesterol/60% lecithin model membranes in the 1970ies gave a leaflet to leaflet distance for the cholesterol headgroup hydroxyls of ~ 39 Å—indicating a thickening of the hydrophobic core by several ångstrøm [92]. The calculated carbonyl to carbonyl distance of ~ 30 Å supports the notion of cholesterol induced membrane thickening. Indeed cholesterol-rich domains are thought to be crucial for the formation of thicker and functionally important raft-domains. Again this underlines that deviation of membrane thickness is connected to specific biological function. A later study by Wiener & White from 1991 that combined X-ray and neutron diffraction of hydrated pure DOPC bilayers by Wiener and White allowed to build a detailed model of the bilayer in the fluid state [93–95]. The high B-factors of the different chemical components that constitute the fluid bilayer such as phosphates, carbonyl groups or water molecules, demonstrated a rather broad Gaussian distribution of their position transversal to the membrane plane, i.e. strong transbilayer thermal motion. Nevertheless, the average carbonyl to carbonyl distance is, as expected, ~ 30 Å. In the universal structural elements of DNA/RNA and proteins, the thickness of an alpha helix or the helical pitch of the DNA double helix is constant dictated by the architecture of the molecular backbone of these biological polymers. In biomembranes, such universal structural elements common to all life forms are missing. Notwithstanding, perhaps the thickness of ~ 30 Å for the hydrophobic core of membranes is a biological constant that might teach us something about the biophysical essence of life. I am not aware of any compelling reason why the hydrophobic thickness of biomembranes should be ~ 30 Å, and not for instance ~ 15 Å or ~ 60 Å. Nor do I know a compelling reason why it appears to be so similar among the many different life forms on our planet while at the same time being composed of such a wide variety of lipid molecules. A possible reason might be evolutionary origin. Perhaps the thickness was fixed by the range of organic molecules available in the chemical environment of submarine hydrothermal vent microstructures for the build-up of the primordial membrane in the Last Universal Common Ancestor [96, 97]. Rapid lateral gene transfer in early life forms of an RNA world [98–100] with bioenergetics based on leaky membranes [101, 102], and a stream of basic hydrogen saturated water, might then have spread a normed transmembrane thickness of the earliest membrane proteins. Or perhaps ~ 30 Å is the minimum thickness that will block the passage of ions, in particular protons, and provide a sufficient stability by the hydrophobic effect, while still allowing a level of compliance compatible with membrane fusion events and sharp membrane bending. It might also be that a hydrophobic thickness of ~ 30 Å is ideal for the generation of electric fields by transmembrane potentials and alpha helical dipole moment. The experimental atomic scale probing of electric fields in biomembranes is a largely unchartered territory that only recently starts to yield to exploration in silico [103–105]. Therefore, geometrical constraints of membrane and membrane protein architecture might be determined more by the physics that govern transmembrane potential fall-off and the resulting electric fields than is clear at present.

**Table 2:** A list of values for the thickness of biomembranes that were assumed or found experimentally

Membrane thickness	Measurement method	Year
33 Å	Bulk electric capacitance of dog red blood cells Fricke [13]	1925
~ 120 Å	Various assumptions Danielli and Davson [14]	1925
~ 45 Å total	Electron crystallography Henderson and Unwin [31]	1975
~ 30 Å core/~50 Å total	Electron crystallography of natural 2D crystals of bacteriorhodopsin Mitsuoka et al. [34]	1999
~ 45 Å total	Electron crystallography of tubular crystals from fish cell membranes Miyazawa et al. [58]	2003
~ 30 Å core/~ 45 Å total	Electron crystallography of sheep lens water channel grown 2D crystals Gonen et al. [36]	2005
~ 25 Å core/~ 40 Å total	X-ray crystallography of 3D crystals of native lipid bilayers containing bacterial rotor rings Murata et al. [60]	2005
~ 28 Å core/~ 38 Å total	Electron crystallography of rat glia cell water channel grown 2D crystals Tani et al. [37]	2009
~ 31–33 Å phosphor to phosphor distance	X-ray crystallography of type I 3D crystals of the SERCA calcium pump from rabbit muscle tissue Norimatsu et al. [81]	2017

The thickness of biomembranes appears to be astonishingly similar across species and type of membrane. Note: used methods of distance measurements are not consistent and the level of precision varies

## Lateral and Transversal Variance

Though the leitmotif of this essay is the by structural methods relative accessible property of membrane thickness, the importance of lateral and transversal heterogeneity has to be mentioned. As described above, it appears that membrane thickness is more uniform than originally anticipated. In contrast, the lateral distribution of lipid species and transversal properties such as lateral pressure seem to be subject to far more variance than suggested by the fluid mosaic model of the biomembrane. A prominent example for lateral deviation from uniformity is lipid rafts [106]. These are cholesterol-rich domains in the lipid bilayer exhibiting lower fluidity and segregation from the more fluid and less ordered areas of the membrane. Lipid rafts are thought to act as specialized functional areas separating and clustering membrane proteins for differing physiological and pathophysiological functions such as signalling or virus budding. Their existence in the living cell and physiological relevance has been a matter of debate mostly due to the fact that good methods for their detection in the living cell were missing. This is a consequence of lipid rafts’ limited size which is in the nanometer range and their dynamic nature which is in the millisecond range [107]. However, the recent advent of super resolution light microscopy provides the field with a tool that can be used to dissect form and functions of lipid rafts in the membranes under physiological conditions [108, 109]. Transversal non-uniformity of the biomembrane is immediately apparent in density profiles of wetted, fluid lipid bilayers which reveal two main areas of approximately equal thickness and radically different hydrophobicity and chemical environment: interfacial headgroup regions and the hydrocarbon core region. An environment far more complex than that of bulk water and crucial for determining the shape of membrane proteins [110]. Variations in the transversal properties of biomembranes as a function of membrane depth such as dipole moment or lateral pressure are even less accessible by direct experimental means than that of lateral variations in lipid composition. As a consequence, despite its biological importance, the property of lateral pressure remains the domain of in silico studies. Its significant variation in dependence on the presence of the non-lamellar lipid DOPE has been demonstrated by computer simulations [111]. These showed an almost constant membrane thickness while lateral pressure in the headgroup region decreased by up to 50% and increased in the lipid tail region by up to ~ 400%. This provides an indication of how lipid composition might modify membrane properties for the regulation of integral membrane protein function. Likewise the effect of general anesthetics has been proposed to be an effect of transversal changes in the lateral pressure profile resulting in a change in the conformational landscape of, e.g. sodium ion channels [112]. This proposal is now corroborated by molecular dynamic simulations which demonstrated that general anesthetics like chloroform, diethylether or enflurane insert into the hydrocarbon core and can cause an increase of lipid area at roughly constant membrane thickness resulting in a decrease in lateral pressure in the headgroup region at the position of the carbonyl groups [113].

## Closing Statement

Cellular membranes are outside of the central dogma of molecular biology. Yet still they are as fundamental to life as are DNA, RNA and proteins. Naturally, the current limitations in our understanding of membrane biology are highly regrettable. That our knowledge on biomembranes lags so far behind that of the other molecules of life is mostly a consequence of the experimental difficulties of dealing with lipid bilayers and membrane proteins. Therefore, it is desirable and anticipated that advances especially in biophysical and computational methods will bring about a more realistic picture of the biomembranes allowing it to come into the place occupied by the fluid mosaic model for already more than 40 years.
